# Genomic selection for QTL-MAS data using a trait-specific relationship matrix

**DOI:** 10.1186/1753-6561-5-S3-S15

**Published:** 2011-05-27

**Authors:** Zhe Zhang, XiangDong Ding, JianFeng Liu, Dirk-Jan de Koning, Qin Zhang

**Affiliations:** 1 Key Laboratory of Animal Genetics and Breeding of the Ministry of Agriculture, College of Animal Science and Technology, China Agricultural University, Beijing, China; 2 The Roslin Institute and Royal (Dick) School of Veterinary Studies, University of Edinburgh, Roslin, UK; 3 Department of Animal Breeding and Genetics, SwedishUniversity of Agricultural Sciences, Uppsala, Sweden

## Abstract

**Background:**

The genomic estimated breeding values (GEBV) of the young individuals in the XIV QTL-MAS workshop dataset were predicted by three methods: best linear unbiased prediction with a trait-specific marker-derived relationship matrix (TABLUP), ridge regression best linear unbiased prediction (RRBLUP), and BayesB.

**Methods:**

The TABLUP method is identical to the conventional BLUP except that the numeric relationship matrix is replaced with a trait-specific marker-derived relationship matrix (TA). The TA matrix was constructed based on both marker genotypes and their estimated effects on the trait of interest. The marker effects were estimated in a reference population consisting of 2 326 individuals using RRBLUP and BayesB. The GEBV of individuals in the reference population as well as 900 young individuals were estimated using the three methods. Subsets of markers were selected to perform low-density marker genomic selection for TABLUP method.

**Results:**

The correlations between GEBVs from different methods are over 0.95 in most scenarios. The correlations between BayesB using all markers and TABLUP using 200 or more selected markers to construct the TA matrix are higher than 0.98 in the candidate population. The accuracy of TABLUP is higher than 0.67 with 100 or more selected markers, which is nearly equal to the accuracy of BayesB with all markers.

**Conclusions:**

TABLUP method performed nearly equally to BayesB method with the common dataset. It also provides an alternative method to predict GEBV with low-density markers. TABLUP is therefore a promising method for genomic selection deserving further exploration.

## Background

With the availability of whole genome high-density single nucleotide polymorphism (SNP) chips in many livestock and plant species, methods using the genomic information to detect the underling architecture of complex traits have become popular. In breeding programmes, the method to predict genomic estimated breeding values (GEBV) with whole-genome markers was termed genomic selection, as proposed by Meuwissen et al. [1]. The general idea of genomic selection is to estimate the effects of dense markers that are distributed across the whole-genome and then sum up the estimated marker effects to obtain the GEBV for genotyped individuals. Many methods have been proposed in the framework of genomic selection [1,2]. In this study, a BLUP method using a trait specific relationship matrix (TA) in the mixed model equations was employed to estimate GEBVs. This method is coined TABLUP [3].

The aim of this study is to validate the TABLUP method and compare it with the ridge regression BLUP (RRBLUP) and the BayesB method using the simulated common dataset provided in the XIV QTL-MAS workshop. We tried to assess the performance of different methods and explain the results either with or without knowing the simulated true breeding values (TBV).

## Methods

### Dataset

The common dataset consists of 3 226 individuals from five consecutive generations (F0 - F4). Each of the 2 326 individuals in generation F0 to F3 has phenotypic records on two traits: a quantitative trait Q and a binary trait B. In this study, we only deal with trait Q. Individuals with phenotypic records (F0 - F3) and without phenotypic records (F4) were treated as reference and candidate population, respectively. A genome consisting of 10 031 biallelic SNPs on 5 chromosomes with 100 million bps length each were simulated without any missing data and genotyping error. All SNPs were included in our analyses.

### Estimation of SNP effects

Both RRBLUP and BayesB were used to estimate SNP effects in the reference population. The statistical model for marker effect estimation can be written as:(1)

where y is the vector of phenotypic values or estimated breeding values; *b* is a vector of fixed effects (including an overall mean); *g_i_* is the random effect of marker *i*; *m* is the total number of markers; *e* is a vector of residual errors; and X and Z_i_ are design matrices corresponding to *b* and *g_i_*. We assumed that residuals *e* are independent and follow a normal distribution, . All marker effects *g_i_* were also assumed to be normally distributed,  for RRBLUP or a scaled inverse chi-square distribution with a proportion of *π* for BayesB.

In RRBLUP, the variance of marker effect was assumed to be identical for all markers and was calculated as , where  is the total additive genetic variance which was estimated from the simulated data. In BayesB, the prior of the proportion of loci without effect, *π*, was estimated from a pre-analysis of the simulated data. The Markov chain was run for 10 000 cycles with 100 cycles of Metropolis-Hastings sampling in each Gibbs sampling, and the first 2 000 cycles were discarded as burn-in. All the samples of marker effects after burn-in were averaged to obtain the marker effect.

### Estimation of GEBVs

The GEBVs of all genotyped individuals were estimated using three methods: TABLUP, RRBLUP and BayesB. For RRBLUP and BayesB, the GEBV of a genotyped individual was calculated as the sum of all marker effects according to its marker genotypes as proposed by Meuwissen et al. [1].

For TABLUP, the GEBVs were estimated based on the following model:(2)

where *y* is the vector of phenotypes of individuals in the reference population and *u* is the vector of breeding values of all genotyped individuals (F0 - F4) with the variance-covariance matrix equal to , where *TA* is a trait specific relationship matrix, and the  was estimated from the reference population via AI-REML and the DMU software [4].

The TA matrix was constructed by using genotypes of all markers and their estimated effects obtained from either RRBLUP or BayesB (denoted as TAP and TAB, respectively), as proposed in our previous study [3]. As an alternative to using all markers, the top 5000, 2000, 1000, 500, 200 and 100 markers were selected from the whole dataset according to the sizes of their effects estimated from the whole dataset to construct the TA matrix.

## Results and discussion

### Variance components

The pedigree and phenotype data of generations F0 - F3 were used to estimate the variance components. The estimated variances are 56.6 for additive genetic effect and 47.7 for residual effect. Therefore, the estimated heritability of trait Q is 0.54.

### Estimates of marker effects

**Figure 1:**
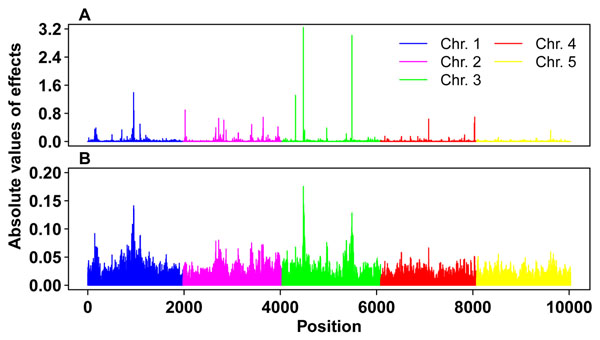
Absolute values of marker effects estimated by BayesB (A) and RRBLUP (B).

Figure 1 shows the marker effects for trait Q estimated by BayesB (Figure 1A) and RRBLUP (Figure 1B). These estimated effects, which are obviously not evenly distributed, reflected the underlying architecture of the trait. Several big QTL were mapped on chromosomes 1 and 3. Markers with large effects should be in high linkage disequilibrium with QTL and could contribute more to the trait than markers in other chromosomal regions. By weighting each allelic relationship between two individuals, the TA matrix not only included the realized relationship but also the genetic architecture of the trait of interest.

### Correlation between GEBVs from different methods

Table 1 shows the correlations between GEBVs from different methods. In general, the GEBVs from different methods are highly correlated with the correlation coefficient over 0.95 in most scenarios, indicating that the GEBVs from different methods are quite consistent. In particular, the correlation between TAB (TABLUP with weights from BayesB) and BayesB is close to 1 in the candidate population. This demostrates the predicting ability of TAB is equal to that of BayesB. However, the lowest correlation between RRBLUP and BayesB indicated that there would be a notable difference in accuracy between them as well as between RRBLUP and TABLUP.

**Table 1:** Correlations between GEBVs from different methods.

	RRBLUP	BayesB	TAP	TAB
RRBLUP		0.989	0.971	0.973
BayesB	0.938		0.982	0.957
TAP	0.985	0.959		1.000
TAB	0.942	0.999	0.963	

Upper triangle and lower triangle show the correlations in the reference population and the candidate population, respectively. TAP and TAB represent TABLUP method using marker effects estimated by RRBLUP and BayesB, respectively.

### TABLUP with low-density markers

**Figure 2:**
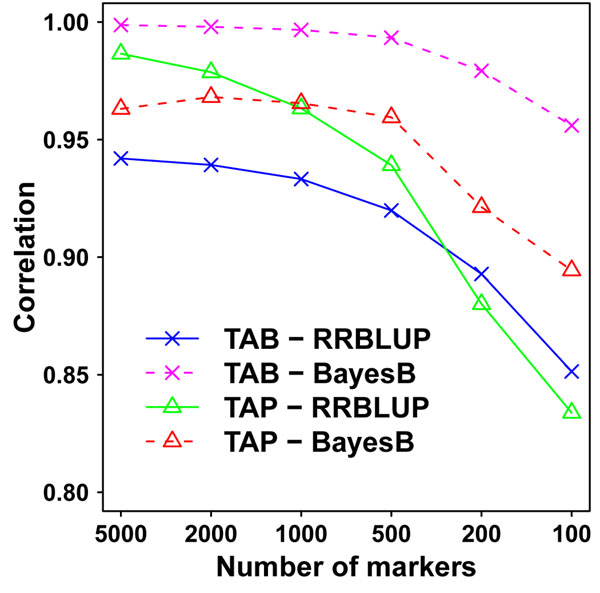
**Correlations of GEBV between TABLUP and RRBLUP or BayesB in candidate population.** The number of markers used in TABLUP ranged from 5000 to 100. The GEBV of RRBLUP and BayesB were estimated from all markers. TAP and TAB represent TABLUP method using marker effects estimated by RRBLUP and BayesB, respectively.

Different subsets of markers were selected based on their size of estimated effects to construct the TA matrix. Figure 2 shows the correlations between GEBVs from TABLUP with different numbers of markers and GEBVs from BayesB or RRBLUP with all markers in the candidate population.

The correlations between TAB and BayesB are always the highest for all numbers of markers. Generally, the correlations increase with the increase of number of markers, but become almost constant when the numbers of markers are over 1 000. Even though only 500 markers (5 percent of all markers) were selected, the correlations of GEBVs between TABLUP and BayesB or RRBLUP are 0.92. In particular, the correlation between TAB and BayesB is close to 1.0 when the numbers of markers are over 500. This implies that TABLUP with only a proportion of selected markers might be recommendable for genomic selection in candidate populations because of the remarkably reduced cost for genotyping, even though there might be a little loss of accuracy.

### Comparison with true breeding values

The availability of true breeding values (TBVs) allowed a more efficient assess of methods. Table 2 shows the correlations of TBVs and GEBVs and regressions of TBVs on GEBVs of different methods. In terms of predicting ability, TAB and BayesB outperformed RRBLUP in this dataset. For TABLUP, the loss of accuracy with low density markers could be neglectable. All methods slightly overestimated the TBVs, except TAP with large number of markers. For TABLUP, the regression coefficient decreases with number of markers included in TA matrix.

**Table 2:** Comparsion with true breeding values

Method	No. marker	*r*	*b*	MSD
BayesB	10031	0.676	0.957	41.9
RRBLUP	10031	0.608	0.943	48.7
TAB	10031	0.675	0.971	42.1
	5000	0.675	0.964	42.1
	2000	0.675	0.950	42.0
	1000	0.677	0.945	41.9
	500	0.678	0.945	41.8
	200	0.675	0.938	42.1
	100	0.672	0.927	43.5
TAP	10031	0.626	1.074	46.9
	5000	0.632	1.045	46.4
	2000	0.640	0.990	45.5
	1000	0.643	0.951	45.3
	500	0.647	0.952	44.9
	200	0.647	0.930	45.0
	100	0.626	0.951	46.9

*r*: correlation coefficient between GEBV and TBV; *b*: regression coefficient of TBV on GEBV; MSD: mean square deviation of (TBV - GEBV) after correcting for mean.

## Conclusions

TheTABLUP method performed comparably to the currently widely used BayesB and RRBLUP methods. It provides the possibility to use low-density markers for estimating GEBV with a relatively high accuracy. It is therefore a promising method for genomic selection and deserves further exploration.

## List of abbreviations used

QTL: quantitative trait locus; MAS: marker assisted selection; SNP: single nucleotide polymorphism; GEBV(s): genomic estimated breeding value(s); TBV(s): true breeding value(s); RRBLUP: ridge regression best linear unbiased prediction; TABLUP: best linear unbiased prediction with trait specific relationship matrix; TAB: TABLUP with weights from BayesB; TAP: TABLUP with weights from RRBLUP.

## Competing interests

The authors declare that they have no competing interests.

## Authors' contributions

ZZ, DXD and LJF carried out the data analyses and contributed the manuscript. QZ and DJK coordinated the analyses and contributed to the manuscript. All authors have read and contributed to the final text of the manuscript.

## References

[B1] Meuwissen THE Hayes BJ Goddard ME Prediction of total genetic value using genome-wide dense marker maps Genetics 2001 157 4 1819 1829 11290733 10.1093/genetics/157.4.1819 PMC1461589

[B2] VanRaden PM Efficient methods to compute genomic predictions J Dairy Sci 2008 91 11 4414 4423 10.3168/jds.2007-0980 18946147

[B3] Zhang Z Liu JF Ding XD Bijma P de Koning DJ Zhang Q Best linear unbiased prediction of genomic breeding values using trait-specific marker-derived relationship matrix PLoS ONE 2010 5 9 e12648 10.1371/journal.pone.0012648 20844593 PMC2936569

[B4] Madsen P Sørensen P Su G Damgaard LH Thomsen H Labouriau R DMU - a package for analyzing multivariate mixed models8th World Congress on Genetics Applied to Livestock Production 2006 Brasil

